# Supplementary material for: Nutritional Supplement with Fermented Soy in Patients Under Active Surveillance for Low-Risk or Intermediate-Risk Prostate Cancer: Results from the PRAEMUNE Trial
Source: Cancers (Basel). 2024 Oct 28;16(21):3634. doi: 10.3390/cancers16213634 (PMC11545628; doi:10.3390/cancers16213634)
Supplement: Supplementary file 1 [file cancers-16-03634-s001.zip › cancers-3231783-supplementary.pdf]

Table S1. Secondary outcomes per response group in the imputed dataset.

	**Responders**	**Non-responders**	**P-value**
Number (%)	101 (55.8%)	80 (44.2%)	
Median age (IQR), years	66 (60-72)	68 (63-72)	0.076
MRI at baseline, n (%)	100 (99.0%)	76 (95.0%)	0.102
PIRADS (highest)			0.171
2	3 (3.0%)	0 (0.0%)	
3	17 (16.8%)	21 (27.6%)	
4	65 (65.3%)	44 (57.9%)	
5	15 (14.9%)	11 (14.5%)	
ISUP at baseline, n (%)			
ISUP 1	90 (89.1%)	63 (78.8%)	0.056
ISUP 2	11 (10.9 %)	17 (21.2%)	
>10% Pattern 4 at baseline, n (%)	0 (0.00%)	0 (0.00%)	
Median (IQR) PSA density at baseline, ng/ml/cm³	0.14 (0.10-0.17)	0.14 (0.10-0.18)	0.553
Median (IQR) total biopsy tumor length at baseline, mm	5 (2-9)	6 (2-10)	0.767
Median (IQR) PSA at baseline, ng/ml	5.9 (4.4-7.8)	6.5 (5.0-9.2)	0.028
Median (IQR) PSA at 3 months , ng/ml	5.3 (3.7-7.1)	6.7 (5.5-9.5)	<0.001
Median (IQR) PSA at 6 months , ng/ml	5.3 (3.3-7.2)	7.6 (6.1-10.7)	<0.001
Median (IQR) PSA at 12months, ng/ml	5.4 (3.5-6.9)	8.7 (7.1-12.2)	<0.001
Median (IQR) PSA at 18months, ng/ml	5.8 (3.8-7.9)	8.6 (7.1-11.3)	<0.001
Control MRI taken anytime, n (%)	86 (85.1%)	73 (91.2%)	0.088
PIRADS			0.476
2	4 (4.7%)	1 (1.4%)	
3	22 (25.6%)	15 (20.5%)	
4	46 (53.5%)	41 (56.2%)	
5	14 (16.3%)	16 (21.9%)	
Control biopsy taken anytime, n (%)	70 (69.3%)	68 (85.0%)	0.014
Control biopsy positive, n (%)	63 (62.4%)	61 (76.3%)	0.048
Control biopsy ISUP (n, %)			
ISUP 1	39 (38.6%)	31 (38.8%)	0.062
ISUP 2	24 (23.8%)	30 (37.5%)	
>10% Pattern 4 at control biopsy (n, %)	19 (18.8%)	21 (26.3%)	0.231
Median (IQR) Pattern 4% at control biopsy, %	30 (20-60)	30 (20-60)	0.893
Median (IQR) total biopsy tumor length at control biopsy, mm	12 (7-20)	11 (5-18)	0.606
Therapy initiated*, n (%)	12 (11.9%)	17 (21.3%)	0.088
Median (IQR) time until therapy, months	18 (16-20)	17 (11-19)	0.055

*Only therapy initiated at the 18 months follow-up (leniency period of 3 months) was taken into account.
